# Higher Performance of DSSC with Dyes from *Cladophora* sp. as Mixed Cosensitizer through Synergistic Effect

**DOI:** 10.1155/2015/510467

**Published:** 2015-01-22

**Authors:** Andery Lim, Noramaliyana Haji Manaf, Kushan Tennakoon, R. L. N. Chandrakanthi, Linda Biaw Leng Lim, J. M. R. Sarath Bandara, Piyasiri Ekanayake

**Affiliations:** ^1^Environmental and Life Sciences Programme, Universiti Brunei Darussalam, Jalan Tungku Link BE1410, Brunei Darussalam; ^2^Physical and Geological Sciences Programme, Universiti Brunei Darussalam, Jalan Tungku Link BE1410, Brunei Darussalam; ^3^Institute for Biodiversity and Environmental Research, Universiti Brunei Darussalam, Jalan Tungku Link BE1410, Brunei Darussalam; ^4^UBD∣IBM Centre, Universiti Brunei Darussalam, Jalan Tungku Link BE1410, Brunei Darussalam; ^5^Chemistry Programme, Universiti Brunei Darussalam, Jalan Tungku Link BE1410, Brunei Darussalam; ^6^Centre for Advanced Material and Energy Sciences, Universiti Brunei Darussalam, Jalan Tungku Link BE1410, Brunei Darussalam

## Abstract

Chlorophyll and xanthophyll dyes extracted from a single source of filamentous freshwater green algae (*Cladophora* sp.) were used to sensitize dye sensitized solar cells and their performances were investigated. A more positive interaction is expected as the derived dyes come from a single natural source because they work mutually in nature. Cell sensitized with mixed chlorophyll and xanthophyll showed synergistic activity with improved cell performance of 1.5- to 2-fold higher than that sensitized with any individual dye. The effect of temperature and the stability of these dyes were also investigated. Xanthophyll dye was found to be more stable compared to chlorophyll that is attributed in the ability of xanthophyll to dissipate extra energy via reversible structural changes. Mixing the dyes resulted to an increase in effective electron life time and reduced the process of electron recombination during solar cell operation, hence exhibiting a synergistic effect.

## 1. Introduction

Dye sensitized solar cells (DSSCs) are photoelectrochemical, alternative energy source devices that convert light energy into electricity, based on the photosensitization of wide-bandgap metal oxide semiconductors such as TiO_2_. Photosensitization is a process whereby energy of absorbed light is transferred from a photosensitizer (light-absorbing molecules such as dyes) to an acceptor (such as semiconductors) [1, 2].

Successfully demonstrated by O'Regan and Grätzel in 1991 [2], the DSSC devices have attracted a lot of interest towards development and improvement of new families of dyes and metal complexes [3]. DSSCs sensitized with dyes having heavy transition-metal complexes such as ruthenium based complex are the most efficient and have been recorded to operate with power conversions efficiency to 11-12% using nanoporous TiO_2_ electrodes [4–6]. However, the high cost of ruthenium complexes and the long-term unavailability of these noble metals [4–8] switch the need to search for alternative photosensitizers to be used in TiO_2_-based photovoltaic devices.

Accordingly, many studies have shown the possibilities of using natural dyes as sensitizers in DSSCs [3, 7]. It is the complexity associated with handling of environmental concerns of using synthetic and metal-based dyes that have encouraged researchers to explore alternative green sources of dyes from various natural resources. The study of natural photosynthesis process has highlighted the functionality of natural pigments in harnessing solar energy [9]. This knowledge has further improved understandings on properties of dye pigments and has shown the pathways to the use of natural dyes or pigments for capturing solar radiation, converting it to more manageable forms of energy. These natural-derived sensitizers, which are commonly involved as light harvesting pigments, offer many advantages over the usage of rare transition metal complexes and other synthetic dyes.

In the nature, these natural pigments serve as antenna complexes that collect light and transfer the energy to “reaction centre complexes” where the chemical oxidation and reduction conversion reactions to long term energy storage take place. These natural pigments are a group of compounds that strongly absorb visible light. Chlorophyll is the well-known and dominant natural pigment in terms of absorbing specific wavelengths of the visible light when harnessing energy from the sun, converting sunlight to chemical energy and transferring of electrons [8]. Others such as carotenoids and anthocyanins are known as accessory pigments that absorb light at different wavelengths than that absorbed by chlorophyll. Besides that they are known to have additional role as protective mechanisms against excess light [1]. It is the energy collectively absorbed by these pigments that drives the light reactions in the process of photosynthesis in higher plants and other photosynthetic organisms.

Such natural pigments can easily be extracted from fruits, vegetables, plants, and flowers with minimal chemical procedures and hence attract a great interest in producing a low cost and yet easy to fabricate DSSC photovoltaic device [10]. Their availability in large quantities in nature, convenient extraction with cheaper organic solvents, ability of application without fine purification, being environmentally friendly, and low production cost of the devices are some of the other competitive advantages of utilizing natural dyes [11, 12].

**Figure 1:**
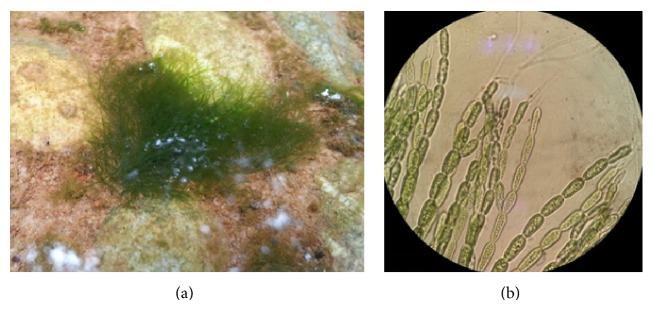
(a)* Cladophora* algae in situ in fresh water pond; (b) light micrograph of* Cladophora* under 1000x magnification.

This paper examines the synergistic performance of chlorophyll and xanthophyll pigments extracted from one type of filamentous freshwater green algae,* Cladophora* sp., as dye sensitizers in DSSCs (Figure 1). Algae are used in the biofuel production and this research would increase its value addition where they could be cultured for their photosensitive pigments. Furthermore, this would highlight the importance of high biodiversity in Brunei Darussalam.

Green algae include unicellular and multicellular colonial flagellates and macroscopic seaweeds that are able to manufacture their own food material through photosynthesis. Factors such as water, light, carbon dioxide, temperature, and minerals are necessary for algal growth [13, 14]. Algae could cope with a highly variable environment such as rapid attenuation of light with depth [13]. Besides, this absorbed light energy could also be reemitted as fluorescence or dissipated as heat rather than being utilized in the photosynthesis [9, 15].

These algal derived photosynthetic pigments are known to work mutually in nature for the photosynthesis process by broadening the light absorption capacity as well as providing photoprotection through xanthophyll cycle which dissipate excess light energy [9, 15]. Furthermore, the photosynthesis process is linked through “Z-scheme” via electron transport coperformed by photosystem I (PSI) and photosystem II (PSII) [16]. In order to increase light absorbing capacity of DSSCs, multiple dye system is desirable. However, mixed dye system would account for many possible types of interactions between dyes with various constituent presents [17].

We hypothesized that, since the pigments were derived from a single species, desirable interactions between dyes could be achieved through synergistic effect in improving electron injection, light harvesting, and limitation of electron recombination. Therefore, this paper discusses our experiment on cosensitization of dyes from a single natural source in evaluating the performance of DSSC using mix chlorophyll and xanthophyll dyes. Stability of the investigated natural pigments under the natural temperature and light intensity variations as well as electron kinetics that exist in the ambient of DSSC was measured.

## 2. Materials and Methods

### 2.1. Extraction of Dye

All extraction procedures were carried out under dim light and glassware containing dyes were covered with aluminium foil to minimize photooxidation. The filamentous freshwater algae of* Cladophora* sp., 20 g, were cleaned and rinsed using distilled water prior to extraction.

The dye pigment was extracted by grinding the algae (20 g) with absolute ethanol (100 mL) and left overnight (refrigerated at about 3°C). The residual solids were filtered off and the filtrate was then centrifuged to separate any remaining solid content and extract containing both chlorophyll and xanthophyll was recovered.

Hot saponification method was adopted to isolate chlorophyll and xanthophyll pigments, by mixing 20% methanolic potassium hydroxide (5 mL) into 50 mL of the recovered extract and kept overnight at 56°C. Petroleum ether (70 mL) was then added and the mixture was shaken and left to stand until two layers of xanthophyll pigment (yellow) and chlorophyll pigment (green) were separated. The yellow coloured layer containing xanthophyll pigment was then washed with acetone in 1 : 1 v/v ratio [18].

The presence of chlorophyll and xanthophyll was confirmed by using the UV-visible absorption spectroscopic techniques (model: SHIMADZU UV-1800).

### 2.2. Assessing Stability of Dye Pigments

Absolute ethanol (4.75 mL) was added to each of the dye pigment (0.25 mL) making a total volume of 5 mL.

The effect of temperature and light were examined at 27°C (laboratory temperature) and 31°C (average temperature under direct sunlight), with light source equivalent to 100 mW/m^2^ illumination. The controls were tightly covered with aluminium foil to protect from exposure to light.

Chlorophyll [19] and xanthophyll [20] concentrations were estimated and converted to percentage retention for accurate comparison among the treatments.

### 2.3. Fabrication of Photoelectrode

TiO_2_ paste Solaronix (nanoxide-T, colloidal anatase particles size: ~13 nm, ~120 m^2^ g^−1^ (BET), Switzerland) was used for photoelectrodes fabrication. The TiO_2_ paste was coated using Doctor Blade method on precleaned fluorine-doped conducting tin oxide (FTO) glasses (~7 Ω sq^−1^). Electrodes were then preheated at ~50°C using a hair-drier and sintered at 450°C for 30 minutes. The thickness of the TiO_2_ electrodes used for this investigation is ~9 *μ*m (Scanning Electron Microscope (SEM)) [21, 22].

### 2.4. Dye Sensitized Solar Cell Preparation and* I-V* Measurements

The TiO_2_ electrodes were subsequently dipped in the extracted chlorophyll, xanthophyll, and mixture of chlorophyll and carotenoid dyes (1 : 1 v/v) for overnight. The electrodes were then rinsed with absolute ethanol and air dried. DSSCs were assembled by introducing the redox electrolyte containing tetrabutylammonium iodide (TBAI; 0.5 M)/*I*_2_ (0.05 M), in a mixture of acetonitrile and ethylene carbonate (6 : 4, v/v) between the dyed the TiO_2_ electrode and platinum counter electrode [21]. Those DSSCs were light soaked by placing the cells under irradiation of 100 mW/cm^2^ for about 4 hours after applying the electrolyte to obtain the best reading. This also allows optimum incorporation of electrolyte into the TiO_2_ layers. The cells were then put under solar simulator (model: DYESOL LP-156B) for current-voltage measurement.

The power conversion efficiency (*η*) was calculated using the following described relation:

(1) $$\begin{array}{c} \eta =\frac{\text{FF}\times I_{\text{sc}}\times V_{\text{oc}}}{P}, \end{array}$$

where *I*_sc_ is the short-circuit photocurrent density (A cm^−2^), *V*_oc_ is the open circuit voltage (V), *P* is the intensity of the incident light (W cm^−2^), and FF is the fill factor defined as FF = *I*_*m*_ *V*_*m*_/*I*_sc_ *V*_oc_, in which *I*_*m*_ and *V*_*m*_ are the optimum photocurrent and voltage that can be extracted from the maximum power calculated from the *I*-*V* data [23, 24].

### 2.5. Electrochemical Impedance Spectroscopic Measurement

Electrochemical impedance spectroscopic measurement was carried out using computer controlled electrochemical interface (SI 1287, Solatron), and impedance/gain-phase analyzer (SI 1260, Solatron). The frequency range and the amplitude of alternative voltage were from 0.01 Hz to 10^5^ Hz and 10 mV, respectively [25]. The impedance measurements were performed at open circuit condition by applying the bias voltage conditions. Impedance parameters and equivalent circuits were then obtained by fitting the spectra with ZView software (v3.3, Scribner Associate Inc.).

## 3. Results and Discussion

**Figure 2:**
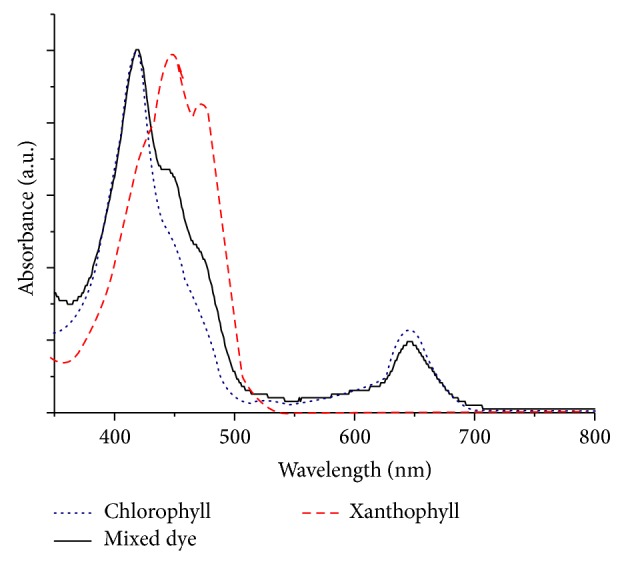
UV-Vis absorption spectra of chlorophyll, xanthophyll, and mixture of chlorophyll and xanthophyll dyes.

**Figure 3:**
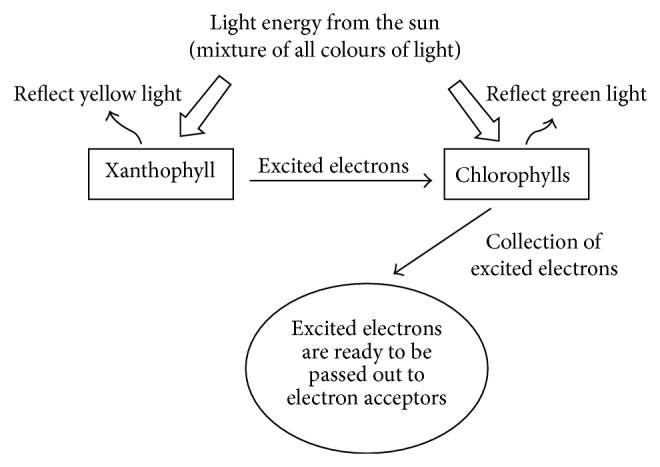
Mutual interaction between xanthophyll and chlorophyll in harvesting of light.

Absorption characteristics of the extracted dyes from filamentous freshwater green algae were recorded using a UV-Vis spectrophotometer. Chlorophyll is an important biomolecule for photosynthesis that absorbs light in the blue and red regions of the visible spectrum.  Figure 2 compares the absorption spectra of chlorophyll, xanthophyll, and dye cocktail (mixture of chlorophyll and xanthophyll, 1 : 1 v/v ratio), where chlorophyll has two absorption peaks at ~420 nm and ~645 nm and xanthophyll is with absorption maxima of ~449 nm. The broader peak at the blue region (~400–500 nm) represents the mixture of chlorophyll and xanthophyll pigments. This broadening can be attributed to the ability of pigment cocktail harvesting a broader spectrum of solar energy that can in turn be used to produce a higher photocurrent. Both pigments are known to be mutually responsible for increasing light harvesting efficiency in the filamentous freshwater green algae. The biological function of xanthophyll is generally accepted as to serve as essential accessory light-harvesting pigments by absorbing photons and transfer them to chlorophyll molecules [26]. Schematic diagram of light harvesting by xanthophyll and chlorophyll is depicted as in Figure 3.

**Figure 4:**
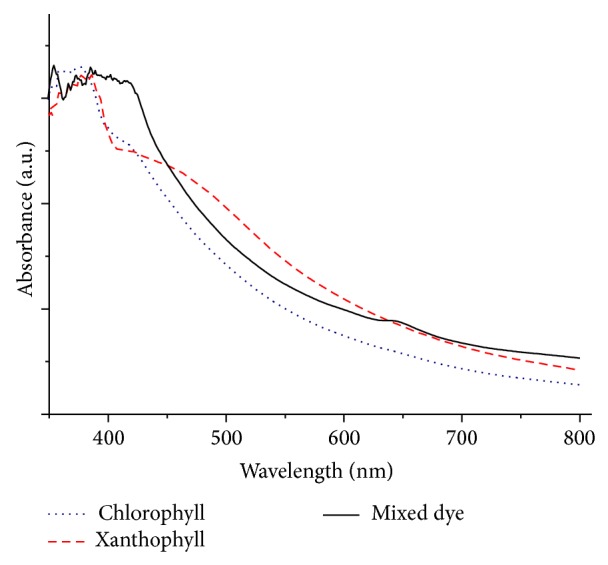
UV-Vis absorption spectra of pigments on TiO_2_ electrode.

The absorption spectra of visible light pertaining to chlorophyll, xanthophyll, and mixed dye pigments (cocktail) on TiO_2_ electrodes are shown in Figure 4. The absorption curves of these spectra are smoother than those shown in Figure 2. Importantly, no obvious absorption peak was observed when these pigments were tested on TiO_2_ electrodes. The absorption of mixed dye was stronger than that of those obtained for chlorophyll and xanthophyll pigments individually. This could be attributed to the interactions between TiO_2_ and the dye molecules on the mesoporous TiO_2_ electrode [16].

Loading of dyes onto TiO_2_ electrodes was investigated by determining the difference in dye concentration before and after dipping the TiO_2_ anode into dye solutions. Concentration of the adsorbed xanthophyll pigments was found to be 2.5 × 10^−4^ *μ*g/mL, much lesser than that of chlorophyll pigments (2.2 *μ*g/mL). Furthermore, it was found that more chlorophyll pigments from the mixed dye system (2.4 *μ*g/mL) could adsorb onto TiO_2_ film than the chlorophyll in the individual dye system. Therefore, a better DSSC performance could be expected from DSSC sensitized using chlorophyll dyes and mixed dyes. Since more chlorophyll from the mixed dye system was adsorbed onto the TiO_2_ electrode, this might indicate that xanthophyll could act as coadsorbent and such synergistic behavior could lead to the enhancement of overall DSSC performance by preventing recombination losses, thus promoting efficient electron injection to take place [27].

**Figure 5:**
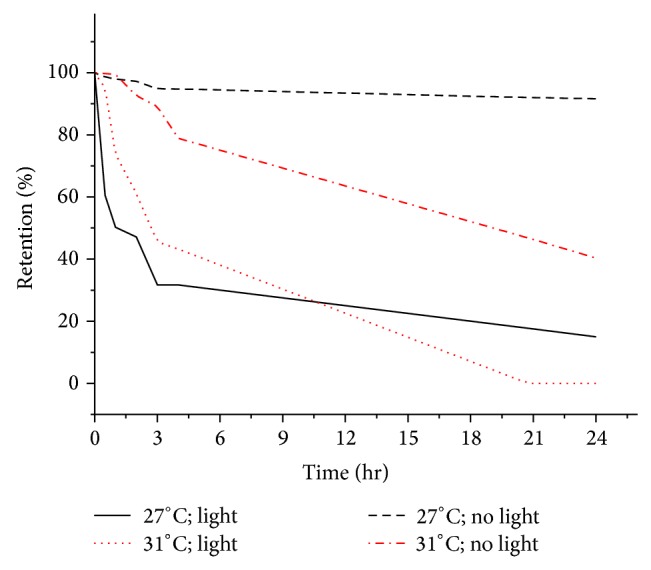
Effect of different temperatures and exposure of light on chlorophyll dye.

Stability of chlorophyll dye exposed to visible light under two different temperatures (27°C and 31°C) expressed in terms of percentage retention are depicted in Figure 5. Chlorophyll dye (with no exposure to light) was found to be more stable at ambient temperature of 27°C. The percentage retention of chlorophyll was reduced from 91.7% to 40.3% when storage temperatures were changed from 27°C to 31°C. The rate of degradation of chlorophyll dye was significantly faster (15% retention at 27°C and zero retention at 31°C over 24 hours period) when exposed to light (100 mW/cm^2^), indicating the higher degree of photosensitivity of this biomolecule. Combination of both high temperature and exposure of light increased the rate of degradation process of chlorophyll suggesting that it is highly susceptible to photodegradation [28].

**Figure 6:**
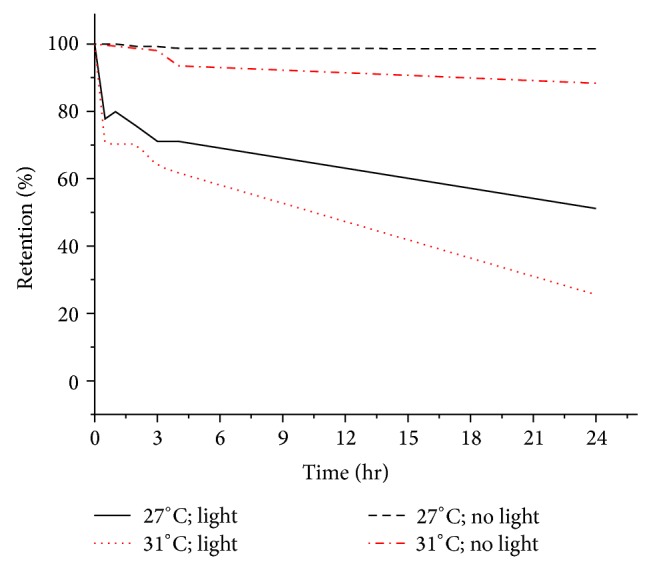
Effect of different temperatures and exposure of light on xanthophyll dye.

As shown in Figure 6, xanthophyll dye was found to be more stable (shows low degradation over 24-hour period) than chlorophyll under light. However, exposure to concurrent high temperature and continuous light made the xanthophyll more prone to photodegradation. The rate of photodegradation of xanthophyll was relatively slower (higher percentage retention over time) than those of chlorophyll (lower percentage retention over time). This confirms the nature of xanthophyll in resisting degradation, thus showing its capability to enhance the light capturing processes in algae and plants. This can be explained by the unique ability of xanthophyll dye that undergoes conformational changes. In nature, xanthophyll pigments exert their photoprotective action by rapidly quenching the excited state of chlorophyll pigments. Xanthophyll has the ability to dissipate excess energy through reversible conformational changes known as the xanthophyll cycle that leads quenching and heat dissipation [9, 29] to protect cell system against photodamage.

In the cycle, three derivatives of xanthophyll change structurally depending on the amount of energy. The conversion of violaxanthin to antheraxanthin and then to zeaxanthin happens under high light conditions. It is known that such conversion is beneficial as zeaxanthin is the most effective of the three xanthophyll derivatives in heat dissipation, where antheraxanthin is only half as effective as zeaxanthin and antheraxanthin make up to 60% of the total xanthophyll cycle pool in photosynthesizing organs that grow under full sunlight conditions, to absorb excess light energy and dissipate as heat thus preventing damage to the photosynthetic activity of chloroplast, which is commonly known as photoinhibition [30].

Therefore, the concurrent effect of high temperature and exposure to light leads to a higher rate of photodegradation of these light-harvesting active biomolecules which could be seen as limiting factors in the usage of natural pigments without structural modification in DSSC.

**Figure 7:**
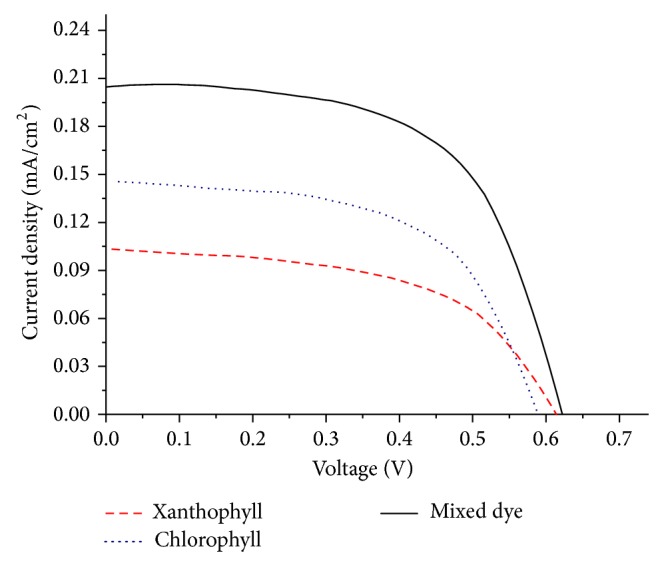
Current-voltage characteristics of the DSSCs using the extracted dyes.

DSSCs fabricated using these extracted dyes were studied to preliminarily determine their synergistic performances as sensitizers of this low cost technology from a “green” source. The current density to voltage (*I*-*V*) characteristics of the DSSCs sensitized with the extracted pigments (Figure 7) is recorded as shown in Table 1.

The performance of the DSSCs sensitized with the chlorophyll dye showed conversion efficiency (*η*) of 0.055%, with open circuit voltage (*V*_oc_) of 0.585 V and short circuit current density (*I*_sc_) of 0.145 mA cm^−2^, and with fill factor (FF) of 0.59. Xanthophyll dye displayed active photochemical activities on the mesoporous TiO_2_ electrode with conversion efficiency (*η*) of 0.038%, open circuit voltage (*V*_oc_) of 0.61 V and short circuit current density (*I*_sc_) of 0.104 mA cm^−2^, and fill factor (FF) of 0.54. In nature, xanthophyll acts as accessory pigments to broaden light harvesting capability. However, the results of this work suggest that xanthophyll too has the ability to absorb light energy and transfers the excited electrons to the semiconductor TiO_2_.

The mixture of chlorophyll and xanthophyll dyes (mix dye) was expected to perform better than individual dyes due to the broadening of the UV-Vis spectrum of mix dye in the blue region (see Figures 2 and 4). The results of power conversion efficiency indicated that mixed dye system performed ~1.5 times and ~2 times higher compared to individual pigment of chlorophyll and xanthophyll, respectively. Similarly, an increase in both short circuit current and open circuit voltage in the mix dye system were observed. This indicates that mixed cosensitization with these two pigments could effectively transfer energy synergistically to the TiO_2_ semiconductor.

In furthering our understanding of the kinetic process of electrochemical and photoelectrochemical processes happening in the DSSC system, electrochemical impedance spectroscopy (EIS) experiment was carried out [25, 30–33]. Electron recombination resistant and effective electron lifetime were determined by fitting EIS spectrum using suitable equivalent circuit that mimics the physical process in the DSSC.

**Figure 8:**
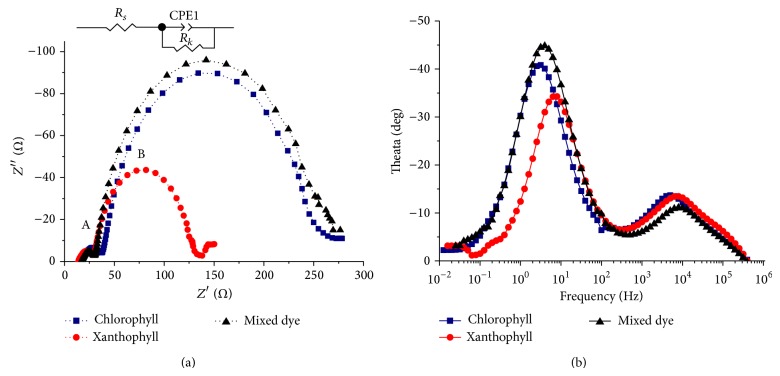
Electrochemical impedance spectra measured with bias voltage conditions and open circuit conditions for DSSCs sensitized with chlorophyll, xanthophyll, and the dye mixture. (a) Nyquist plots with an inset showing the equivalent circuit diagram. (b) Bode phase plot.

Figure 8(a) shows Nyquist plots of DSSCs sensitized with individual chlorophyll, xanthophyll, and mix dyes, under bias voltage conditions. The first arc (A) corresponds to the impedance at Pt electrode/electrolyte and the second arc (B) corresponds to the impedance at TiO_2_/dye/electrolyte interfaces. These impedances were determined by fitting the EIS data in terms of an appropriate equivalent circuit as shown as an inset in Figure 8(a). The magnitude of the second arc of the Nyquist plot can be used to deduce the charge transfer resistances related to the recombination of electrons (*R*_*k*_) in TiO_2_/dye/electrolyte interface [30]. The *R*_*k*_ values were 205 Ω, 97 Ω, and 222 Ω for DSSCs sensitized with chlorophyll, xanthophyll, and mixed dye, respectively. Higher *R*_*k*_ value corresponds to a lower probability in the recombination of electrons. Low recombination resistance (*R*_*k*_) in TiO_2_/dye/electrolyte interface directly affects decay of *V*_oc_ in DSSCs due to high recombination process [25]. Energy transfer between chlorophyll and xanthophyll molecules via quenching effect could reduce the recombination of dark current [16]. Such interactions could also positively contribute to the overall durability of the DSSC.

Figure 8(b) illustrates the Bode plot of chlorophyll, xanthophyll, and mixed dye sensitized DSSCs. Peaks at the low frequency (10^−1^–10^2^ Hz) and the high frequency (10^3^–10^5^ Hz) were associated with effective life-times of electrons on the interface of TiO_2_/dye/electrolyte (*τ*_eff_) and on the platinum electrode/electrolyte interface, respectively. These peak frequencies were inversely proportional to the respective effective life-times of electrons [30]. *τ*_eff_ was calculated for chlorophyll, xanthophyll, and mixed dye sensitized DSSC as 0.313, 0.123, and 0.250 seconds, respectively (see Table 2).

These results indicate a higher effective life-time of electrons (*τ*_eff_) when DSSC was sensitized with chlorophyll and mixed dye as compared to that of xanthophyll. Mixed dye with the highest electron recombination resistance (*R*_*k*_) in TiO_2_/dye/electrolyte interface performed best among the tested DSSCs. In order to obtain an efficient DSSC, a reduction in charge transport resistance (*R*_*w*_), while increase in the combined factors such as effective life-time of electrons (*τ*_eff_), electron recombination resistance (*R*_*k*_), electron density (*ɳ*_s_) and diffusion factor (*D*_eff_), in TiO2/dye/electrolyte interface is required. Therefore, the efficiency of DSSC sensitized with mixed dye was in the agreement with the electrochemical impedance data. The results revealed that the synergistic effect of chlorophyll and xanthophyll dominantly increased the electron recombination resistance and therefore enhanced the photoconversion efficiency of DSSC.

## 4. Conclusions

Dyes derived from a single natural source,* Cladophora* algae, give positive interaction when employed, as a mix, in DSSC as the sensitizer. The extracted chlorophyll and xanthophyll were used as mixed dye sensitizer to obtain an enhanced overall cell performance whereby the *η* was increased up to 1.5 and 2 times as compared to the respective individual dyes. Both temperature and light were found to be critical factors in the degradation of both chlorophyll and xanthophyll dyes. Photodegradation was best indicated by individual chlorophyll dye, where total degradation was observed in less than 24 hours at higher temperature and the presence of light. However, quenching effect performed by xanthophyll through conformational changes (xanthophyll cycle) could contribute to the overall durability of the DSSC. The synergistic performance of the mixed dye in the DSSC photovoltaic system resulted in a reduction in electron recombination (high electron recombination resistant) process that contributed to increase in the overall cell performance.

## Figures and Tables

**Table 1:** The current-voltage performance of DSSCs sensitized by individual chlorophyll, xanthophyll, and mixture of chlorophyll and xanthophyll (mix dye).

Dye sensitizer	*V*_oc_ (V)	*I*_sc_ (mA/cm²)	FF	Efficiency (%)
Chlorophyll	0.585	0.145	0.59	0.055
Xanthophyll	0.610	0.104	0.54	0.038
Mix dye (1 : 1 v/v ratio)	0.619	0.206	0.60	0.085

**Table 2:** Electrochemical impedance spectroscopic parameters of the studied DSSCs.

Dye sensitizer	R_k_ (Ω)	τ_eff_ (s)	R_w_ (Ω)	*D*_eff_ (cm^2^/s) (10^−9^)	ɳ_*s*_ (10^29^)	η (%)
Chlorophyll	205	0.313	10.25	5.18	1.5	0.055
Xanthophyll	97	0.123	3.46	17.9	0.9	0.038
Mix dye (1 : 1 v/v ratio)	222	0.250	8.88	8.1	1.2	0.085
